# The Volatile Oil of Nardostachyos Radix et Rhizoma Induces Endothelial Nitric Oxide Synthase Activity in HUVEC Cells

**DOI:** 10.1371/journal.pone.0116761

**Published:** 2015-02-02

**Authors:** Maitinuer Maiwulanjiang, Cathy W. C. Bi, Pinky S. C. Lee, Guizhong Xin, Abudureyimu Miernisha, Kei M. Lau, Aizhen Xiong, Ning Li, Tina T. X. Dong, Haji A. Aisa, Karl W. K. Tsim

**Affiliations:** 1 Division of Life Science and Centre for Chinese Medicine, The Hong Kong University of Science and Technology, Hong Kong, China; 2 Xinjiang Key Laboratory of Plant Resources and Natural Products Chemistry, Xinjiang Technical Institute of Physics and Chemistry, Chinese Academy of Sciences, Urumqi, China; The Chinese University of Hong Kong, HONG KONG

## Abstract

Nardostahyos Radix et Rhizoma (NRR; the root and rhizome of *Nardostachys jatamansi* DC.) is a widely used medicinal herb. Historically, NRR is being used for the treatment of cardiovascular and neurological diseases. To search for active ingredients of NRR, we investigated the vascular benefit of NRR volatile oil in (i) the vasodilation in rat aorta ring, and (ii) the release of nitric oxide (NO) and the phosphorylation of endothelial NO synthase (eNOS) in cultured human umbilical vein endothelial cells (HUVECs). By measuring the fluorescence signal in cultures, application of NRR volatile oil resulted in a rapid activation of NO release as well as the phosphorylation of eNOS: both inductions were markedly reduced by L-NAME. In parallel, the phosphorylation level of Akt kinase was markedly increased by the oil treatment, which was partially attenuated by PI3K/Akt inhibitor LY294002. This inhibitor also blocked the NRR-induced NO production and eNOS phosphorylation. In HUVECs, application of NRR volatile oil elevated the intracellular Ca^2+^ level, and BAPTA-AM, a Ca^2+^ chelator, reduced the Ca^2+^ surge: the blockage were also applied to NRR-induced eNOS phosphorylation and NO production. These findings suggested the volatile oil of NRR was the major ingredient in triggering the vascular dilatation, and which was mediated via the NO production.

## Introduction

Nardostachyos Radix et Rhizoma, the root and rhizome of *Nardostachys jatamasi* DC., is widely distributed in hilly areas of China, India, Pakistan and Nepal [1–3]. The water decoction of NRR has been used as folk medicine for the treatment of cardiovascular and neuronal diseases [4–6]. In Xinjiang of China, NRR is frequently used as a herbal decoction for cardiovascular disease, e.g. arrhythmia, coronary heart diseases and atherosclerosis [7–8]. In traditional Uyghur medicine, Song Bu Li decoction is prepared from water extract and volatile oil of NRR, and this herbal decoction is mainly used for cardiovascular dysfunction [9]. NRR is highly rich in volatile oil, about 2% by total weight [6], and which plays synergistic role with NRR water extract in activities of neuroprotection [6]. In addition, NRR volatile oil was shown to protect cultured H9c2 cardiomyocytes from oxidative stress-induced cell death [10]. However, the mechanisms by which the volatile oil exerts its role in cardiovascular and circulation system are unknown.

The endothelial dysfunction is the major step in a chain of events that leads to atherosclerosis and coronary heart disease. Endothelial cell dysfunction together with low levels of nitric oxide (NO) and endothelium-derived relaxation factor were found in the causes of atherosclerosis [11]. The endothelium is a thin layer of cell that lines the interior surface of blood vessels, forming an interface between blood cell and blood vessel. The endothelium of blood vessel released NO to signal the surrounding smooth muscle to relax for increasing blood flow [12]. NO is synthesized endogenously from L-arginine, oxygen and NADPH by endothelial NO synthase (eNOS): the NO production is governed by multiple signaling, including the regulation of eNOS expression and phosphorylation [13]. The phosphorylation at Ser^1177^ of eNOS plays an important role in the regulation eNOS activity and NO production [14], and PI3K/Akt signaling is able to phosphorylate eNOS and increase NO production [15]. On the other hand, an increased intracellular free Ca^2+^ could activate eNOS via a Ca^2+^/CaM-dependent protein kinase (CaM kinase). Similar to Akt phosphorylation, CaM kinase could mediate the rapid activation of eNOS and vasodilation [16]. Here, we aimed to reveal the signaling triggered by NRR volatile oil in cultured human umbilical vein endothelial cells (HUVECs), including (i) the activation of NO production and eNOS phosphorylation; (ii) the phosphorylation of Akt kinase; and (iii) the surge of intracellular Ca^2+^.

## Materials and Methods

### Plant materials and chemicals

NRR, the root and rhizome of *N*. *jatamansi*, was purchased from Hong Kong herbal market. No specific permission was required for this action. In addition, NRR is not classified as an endangered species. The authentication of plant material was performed by Dr. Tina T. X. Dong according to their morphological characteristics. The corresponding vouchers for NRR, as forms of the whole plant, were deposited in Center for Chinese Medicine, Hong Kong University of Science and Technology. 3-(4,5-dimethylthiazol-2-yl)- 2,5-diphenyl tetrazolium bromide (MTT), A23187, L-NAME, BAPTA-AM (all at >98% purity) were purchased from Sigma Chemical Co. (St. Louis, MO). LY294002 was purchased from Cell Signaling Technologies (Danvers, MA), Ultra-pure water was prepared from a Milli-Q purification system (Millipore, Molsheim, France).

### Preparation of NRR volatile oil

The total volatile oil from NRR was obtained by water distillation method. Briefly, 50 g of NRR herb was minced and soaked in Milli-Q water in proportion of 1:10 (w/v) overnight. The mixture was submitted to hydro-distillation in a Clevenger-type apparatus for 4 hours. Volatile oil was dried over anhydrous sodium sulfate. The yield of volatile oil was ~1 mL (~2%, v/w), and the oil was stored at -20°C until analyze. For cell culture, the volatile oil stock solution was prepared in DMSO solution with the concentration of 180 mg/mL. The volatile oil solution was further diluted with cell culture medium during the cell experiments.

### Artery preparation

Male Sprague–Dawley rats (~250–300 g) were sacrificed by cervical dislocation and bled. The thoracic aorta was excised. After surrounding connective tissue had been carefully cleaned off, four 3-mm-wide and 2-mm-long ring segments were prepared from each aorta. The rings were suspended between two stainless wire hooks in a 10-mL organ bath. The upper wire was connected to a force–displacement transducer (Grass Instruments, Rockland, MA), and the lower one was fixed at the bottom of organ bath. The organ bath was filled with Krebs solution of the following composition: 119 mM NaCl, 4.7 mM KCl, 25 mM NaHCO_3_, 2.5 mM CaCl_2_, 1 mM MgCl_2_, 1.2 mM KH_2_PO_4_, and 11 mM D-glucose. The bathing solution was gassed with 95% O_2_–5% CO_2_ at 37°C (pH 7.4). The rings were placed under an optimal basal tone of 20 mN, determined from previous length–tension experiments. Changes in isometric tension were measured with a Grass force transducer and stored on MacLab software (Version 3.0, AD Instruments, Colorado Spring, CO) for later data analysis. Twenty minutes after mounting in organ baths, the rings were first contracted with 0.5 µM phenylephrine to test the contractility and then relaxed by the application of ACh. They were rinsed several times until baseline tone was restored. The rings were thereafter allowed to equilibrate for 60 min. Baseline tone was re-adjusted to 20 mN when necessary. Each set of experiments was performed on rings prepared from different rats. The use of laboratory animals was approved by the Animal Research Ethical Committee.

### Cell cultures and viability assay

HUVEC cells were obtained from American Type Culture Collection (ATCC, Manassas, VA) and cultured on 0.2% gelatin-coated T75 flask maintained in culturing medium (M199) supplemented with 20% fetal bovine serum, 90 mg/mL heparin sodium salt, 20 µg/mL endothelial cell growth serum (ECGS), 100 U/mL penicillin and 100 µg/mL streptomycin, at 37°C in a water-saturated 5% CO_2_ incubator. HUVEC cells between passage 3 and 8 were used in these studies as to ensure the genetic stability of the culture [17]. All culture reagents were from Invitrogen (Carlsbad, CA). HUVEC cells were plated in a 96-well plate and treated with volatile oil in series of concentrations for 24 hours. Then, cell viability test was performed with the addition of thiazoly blue tetrazolium bromide (MTT) in PBS at a final concentration of 0.5 mg/mL for 3 hours. After the solution was removed, the purple precipitate inside the cells was re-suspended in DMSO and then measured at 570 nm absorbance [18]. The percentage of cell viability was calculated compared with untreated control.

### Laser confocal fluorescence microscopy

Fluorimetric measurements were performed on cultured HUVEC cells using an Olympus Fluoview FV1000 laser scanning confocal system (Olympus America Inc., Melville, NY) mounted on an inverted l X 81 Olympus microscope, equipped with a 10X objective. Intracellular NO production and calcium concentration were monitored using fluorescent NO indicator 4-amino-5-methylamino-20, 70-difluorofluorescein (DAF-FM DA, Invitrogen, Grand Island, NY) and fluorescent calcium indicator Fluo-4 AM (Invitrogen), respectively. DAF-FM DA reacted not with NO itself but with NO^+^ equivalents, such as nitric anhydride (N_2_O_3_), which were formed by autoxidation of NO. Cultured HUVEC cells, seeded on the glass coverslips, were incubated for 30 min at 37°C in normal physiological solution containing 1 µM DAF-FM DM or Ca^2+^ free normal physiological solution containing 5 µM Fluo-4 AM. The amount of NO or Ca^2+^ were evaluated by measuring the fluorescence intensity excited at 495 nm and emitted at 515 or exited at 488 nm and emitted at 525 nm, respectively. Changes in intracellular NO production and Ca^2+^ were displayed as a ratio of fluorescence relative to the intensity (Fn/F0). Normal physiological saline solution (NPSS) contained 140 mM NaCl, 5 mM KCl, 1 mM CaCl_2_, 1 mM MgCl_2_, 10 mM glucose and 5 mM HEPES (pH 7.4), while Ca^2+^ free solution contained 140 mM NaCl, 5 mM KCl, 1 mM MgCl_2_, 10 mM glucose, 5 mM HEPES, pH7.4 and 0.22 mM EGTA [17].

### Determination of phosphorylation

The phosphorylation of eNOS and Akt in HUVEC cells were determined by western blot assay. The cultures were serum-starved with or without the inhibitors for 3 hours before the drug applications. After drug treatments, the cultures were collected immediately in lysis buffer (125 mM Tris-HCI, 2% SDS, 10% glycerol, 200 mM 2-mercaptoethanol, pH 6.8), and the proteins were subjected to SDS-PAGE analysis. Proteins were separated on the 8% SDS-polyacrylamide gels and transferred to the nitrocellulose membrane. Transfer and equal loading of samples was confirmed by staining Ponceau-*S*. The nitrocellulose membrane was blocked with 5% fat-free milk in TBS-T (20 mM Tris base, 137 mM NaCl, 0.1% Tween-20, pH 7.6) for 2 hours at room temperature. Phosphorylated eNOS and Akt were recognized by anti-phospho-eNOS antibody (1:2000) and anti-phospho-Akt antibody (1:5000; Cell Signaling, Danvers, MA) at 4°C for 16 hours, and horseradish peroxidase (HRP)-conjugated anti-rabbit secondary antibody (1:5000; Invitrogen) for 2 hours at room temperature. The immune complexes were visualized using the enhanced chemiluminescence (ECL) method (GE Healthcare, Piscataway, NJ). The intensities of the bands in the control and different samples, run on the same gel and under strictly standardized ECL conditions, were compared on an image analyzer, using a calibration plot constructed from a parallel gel with serial dilutions of one of the samples [17, 19].

### Other assays

Statistical test was done by using one-way ANOVA. The data was separated into two groups to compare both activation and drugs over the control. The control group was varied in different experiments, and which was specified in the figure legends. Data were expressed as Mean ± SEM, where *n* = 3 to 5. Statistically significant changes were classed as significant [*] where p<0.05, highly significant [**] where p<0.01.

## Results

### NRR volatile oil induces vasodilation in rat aorta ring

**Figure 1:**
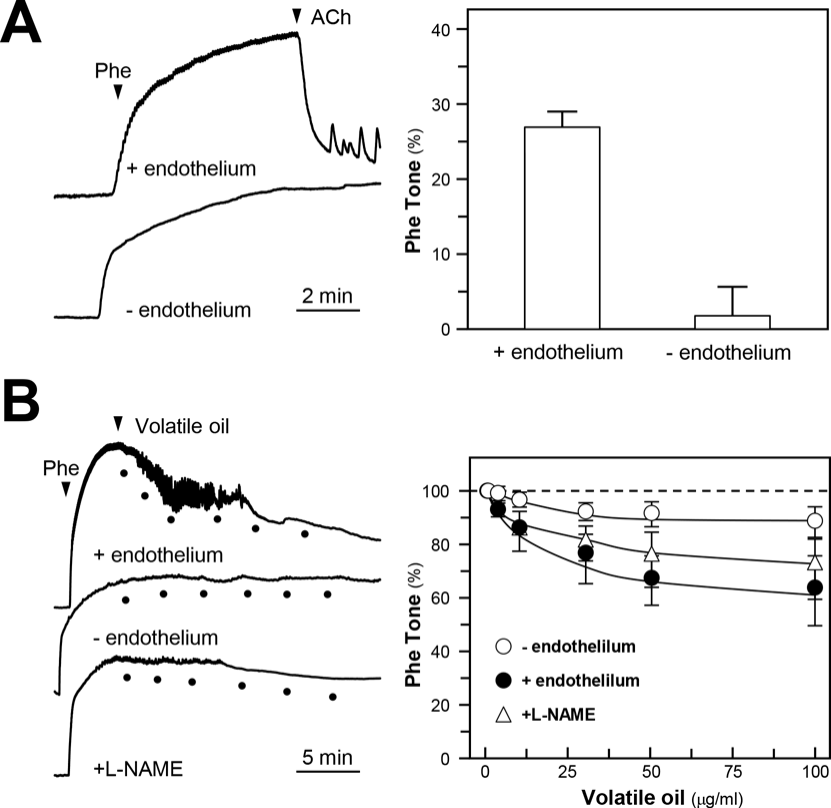
NRR volatile oil induces vasodilation of rat aortic ring. **(A):** Rat aortic ring was isolated with or without intact endothelium, the vasoconstriction was induced by the applied phenylephrine (Phe, 0.5 µM); acetylcholine (ACh, 1 µM) was then added (left panel). **(B):** The contraction of aortic ring was tested similar to (A). Different concentrations of NRR volatile oil (1, 3, 10, 30 and 100 µg/mL) were added to induce the relaxation. Also, L-NAME (100 µM) was applied for 30 min, and then different concentrations of NRR volatile oil were added. Values are expressed as percentage of Phe tone as comparing to the control resting tension (right panel). Mean ± SEM, *n* = 3.

The endothelial cells line the entire circulatory system, including the heart to the smallest capillary, which play a key role as sensor of chemical and physical stimuli. The endothelial cells possess the ability to release NO, a critical mediator for vasodilation in blood vessels. In an isolated rat aortic ring, acetylcholine (ACh) was shown to induce the endothelium-dependent relaxations, and the relaxations were abolished by removing the endothelium (Fig. 1A). The representative traces in Fig. 1B showed that phenylephrine (Phe), a vasoconstrictor, produced contraction effect in rat aorta. The Phe-induced contraction effects could be relaxed by application of NRR volatile oil (Fig. 1B). The NRR volatile oil-induced vasodilation could be reduced by pre-treatment of eNOS blocker L-NAME: this effect could be attenuated by removing the endothelium (Fig. 1B). These results indicated that the NRR volatile oil-induced rat aorta ring relaxation was mediated by NO, produced by endothelial cells.

### NRR volatile oil induces production of NO via phosphorylation of eNOS in HUVECs

NRR volatile oil was prepared according to the optimized extraction methods as described previously [6]. In order to standardize the volatile oil chemically, we analyzed the chemical compositions of NRR volatile oil by GC-MS. The major chemical compositions and its relative amounts were listed. Calarene was the major ingredient, over 35% of total oil. The standardized NRR volatile oil should contain at least the amounts of chemicals as described in Table 1. From the calculations of extraction efficiency, the yield of total volatile oil was 2.1 ± 0.28% (*n* = 4). The established chemical parameters served as a control for repeatability of the below biochemical analyses.

**Table 1:** Major chemical composition of volatile oil from NRR.

Compound^a^	RA (%)^b^	Compound^a^	RA (%)^b^
β-Maaliene	7.9	α-Maaliene	1.6
9-Aristolene	4.7	Patchouli alcohol	5.5
Calarene	38	Guaina-6,9-diene-4 β ol	3.7
Valerena-4,1(11)-diene	6.6	Aristolone	2.1
β-lonone	2.1	Unknown	27.8

^a^ The identified constituents are listed.

^b^ RA indicates relative amount (peak area relative to the total peak area).

The extraction efficiency of NRR oil was over 95% within the 4 hours or distillation. In addition, the amount of extractive oil was 2.1 ± 0.28% (*n* = 4). The values are in mean of three individual experiments (*n* = 3). The SD values were less than 5% of the mean, not shown for clarity.

HUVEC cells have been widely used to study the endothelium dysfunction and vasodilation. Constitutive NO synthesis was found in HUVEC cells [14]. Here, HUVEC cells were applied to study the role of NRR volatile oil on induction of NO production. Cultured HUVECs were treated with series concentration of NRR volatile oil, and MTT assay was performed to assess cell viability. NRR volatile oil from the doses of 3 to 25 g/mL did not have cytotoxicity. Very marginal cell proliferation was found after treatment from 6 to 25 g/mL; however, the cell viability was markedly decreased at higher doses of 50 to 100 g/mL (S1 Fig).

**Figure 2:**
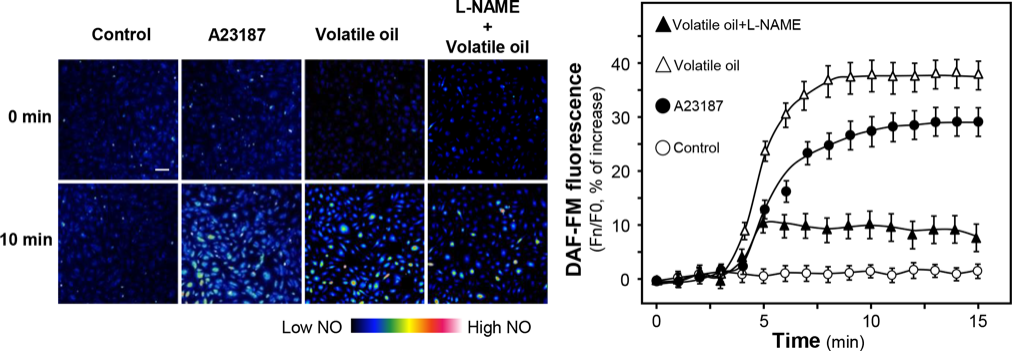
NRR volatile oil stimulates the production of NO in cultured HUVECs. Cultured HUVECs were pre-treated with serum free medium or L-NAME (100 µM) for 3 hours, and then labeled with fluorescent NO indicator DAF-FM DA for 30 min. Then the cells were washed with 1X NPSS at pH = 7.4, and then fluorimetric measurement were performed after the treatment of volatile oil (25 µg/mL), A23187 (1 µM, positive control), or control (without drug treatment). The amounts of NO were evaluated by measuring the fluorescence intensity excited at 495 nm and emitted at 515 nm. Micrographs were taken by the confocal microscope. Bar = 100 µm. (left panel). Quantification of intracellular NO production was displayed as a ratio of fluorescence intensity at any time (Fn) to the control at time 0 (F0) in the cultures (right panel). Mean ± SEM, *n* = 4.

**Figure 3:**
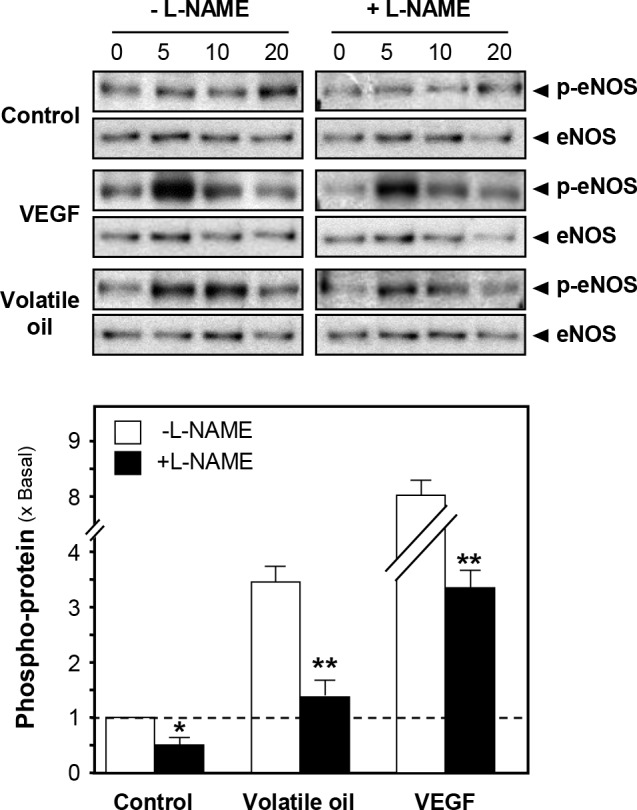
Phosphorylation of eNOS by NRR volatile oil in cultured HUVEC cells. Cultured HUVECs were pre-treated with serum free medium or L-NAME (100 µM) for 3 hours, and treated with NRR volatile oil (25 µg/mL), VEGF (10 ng/mL, positive control) or control (serum free medium) for different time points (0 to 20 min). Phospho-eNOS Ser^1177^ (~135 kDa) and total eNOS (~135 kDa) were revealed by using specific antibodies (upper panel). Quantification of phospho-eNOS protein (at 5 min) from the blot was calculated by a densitometer (lower panel). Data were expressed as x Basal where the control (untreated culture) was set as 1, Mean ± SEM, *n* = 4, each with triplicate samples. **p<0.01.

The production of NO in HUVEC cells was determined after treatment of NRR volatile oil. Endogenous NO production was monitored using specific dye DAF-FM DM. Application of NRR volatile oil triggered a progressive rise in intracellular NO production in cultured HUVECs, as reflected by an increase of fluorescence intensity, which peaked at around 8 min after the drug treatment (Fig. 2). A23187, a calcium ionophore, was employed as a positive control to evoke NO production (Fig. 2). The maximal NO production was better in the scenario of NRR oil treatment. Additionally, L-NAME, an eNOS blocker, also markedly attenuated the volatile oil-mediated NO production in HUVEC cells (Fig. 2), suggesting that NRR volatile oil-induced NO production was mediated by eNOS in HUVECs. To investigate the role of eNOS and its upstream regulators, the phosphorylation of eNOS was first determined to reflect the eNOS activity. The phosphorylation of eNOS at Ser^1177^ (~135 kDa) in HUVECs was observed by 5 min treatment of the oil. Vascular endothelial growth factor (VEGF), a positive control, markedly elevated the phosphorylation of eNOS by ~8 folds (Fig. 3). The phosphorylation of eNOS after treatment of VEGF and NRR volatile oil was reduced by pre-treatment of L-NAME in HUVECs (Fig. 3).

### PI3K/Akt signaling pathway in eNOS phosphorylation by NRR volatile oil

**Figure 4:**
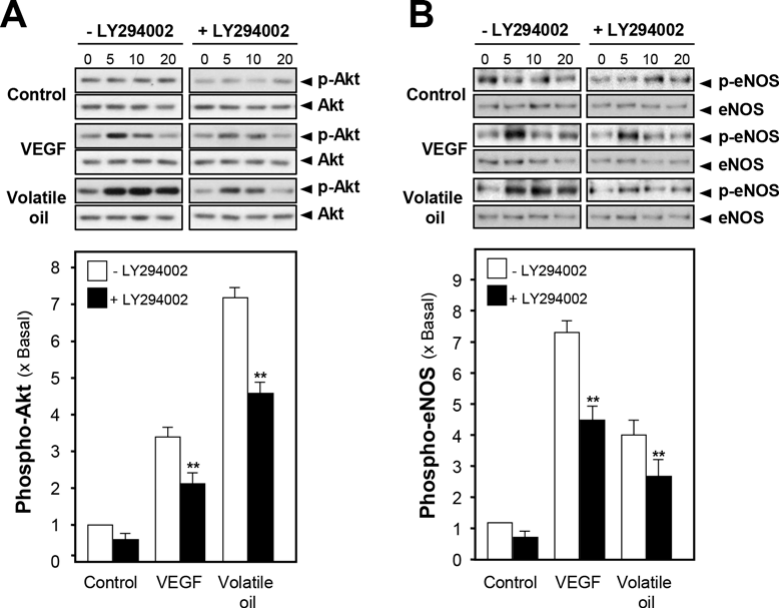
NRR volatile oil-induced eNOS phosphorylation is mediated by PI3K/Akt signaling pathway. Cultured HUVECs were pre-treated with serum free medium or LY294002 (3 µM) for 3 hours, and treated with NRR volatile oil (25 µg/mL), VEGF (10 ng/mL, positive control) or control (serum free medium) for different time points (0–20 min). The cell lysates were obtained for western blotting. **(A)** Phospho-Akt Ser^473^ (~60 kDa) and total Akt (~60 kDa) were revealed by using specific antibodies. **(B)** Phospho-eNOS Ser^1177^ (~135 kDa) and total eNOS (~135 kDa) were revealed. The quantification from the blot in (A) and (B) was performed by a densitometer (lower panel). Data were expressed as x Basal where the control was set as 1. Mean ± SEM, *n* = 3, each with triplicate samples. **p<0.01.

**Figure 5:**
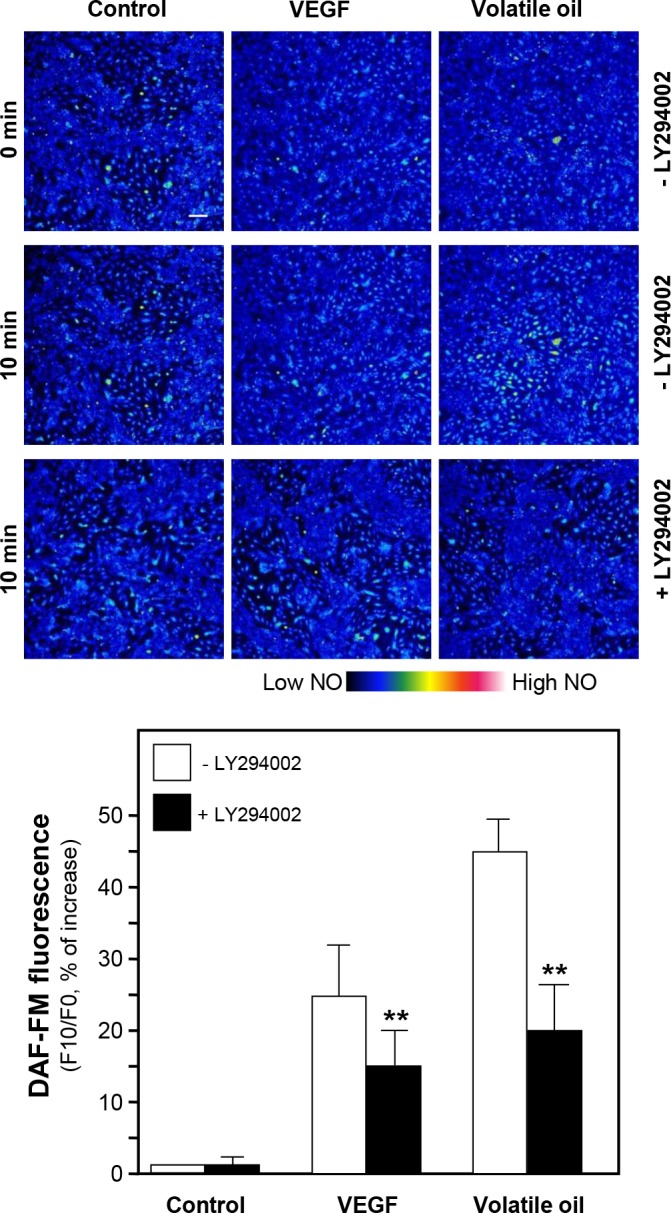
Volatile oil-induced NO production is blocked by LY294002. Cultured HUVECs were pre-treated with serum free medium or LY294002 (1 µM) for 3 hours, and then labeled with fluorescent NO indicator DAF-FM DA for 30 min. Fluorimetric measurement was performed after the treatment of NRR volatile oil (25 µg/mL), VEGF (100 ng/mL, positive control). The amounts of NO were evaluated by measuring the fluorescence intensity (upper panel). Micrographs were taken by the confocal microscope. Bar = 100 µm (upper panel). Quantification of NO production was displayed as a ratio of fluorescence intensity at 10 min (F10) to the control at time 0 (F0) in the cultures (lower panel). Mean ± SEM, *n* = 3, each with triplicate samples. **p<0.01.

The activated PI3K/Akt signaling leads to phosphorylation of eNOS and increases the production of NO [15]. Here, the phosphorylation of Akt in NRR volatile oil-treated HUVECs was evaluated by using specific antibodies, i.e. phospho-Akt Ser^473^ (~60 kDa) and total Akt (~60 kDa). The phosphorylation of Akt was peaked, at ~7 folds, after the treatment of NRR volatile oil after 5 min. VEGF increased the phosphorylation of Akt by ~3 folds (Fig. 4A). In order to investigate possible connection between Akt pathway and eNOS phosphorylation, HUVEC cells were pre-incubated with a kinase-specific blocker, LY294002, before the application of NRR volatile oil. After the treatment, the cell lysates were subjected to analyze phosphorylation level of Akt Ser^473^ and eNOS Ser^1177^. Total Akt and total eNOS were analyzed for normalizing the phospho-proteins. The pre-treatment of LY294002 significantly reduced the phosphorylation level of Akt in volatile oil-treated HUVEC cultures (Fig. 4A). In addition, the phosphorylations of eNOS in NRR volatile oil- and VEGF-treated cultures were significantly reduced by pre-treatment of Akt inhibitor LY294002 (Fig. 4B). In both cases, total protein levels of Akt and eNOS were not altered. To confirm the role of Akt pathway in NRR volatile oil-mediated NO production in HUVECs, LY294002 was applied on HUVECs for 3 hours before determination of NO production after the treatment of volatile oil. Pre-treatment of LY294002 suppressed VEGF and volatile oil-mediated NO production in HUVEC cells (Fig. 5). These results suggested the possible connection between Akt signaling pathway and the volatile oil-mediated NO production in cultured HUVEC cells.

### Ca^2+^/CaM signaling pathway in NRR volatile oil-mediated eNOS phosphorylation

**Figure 6:**
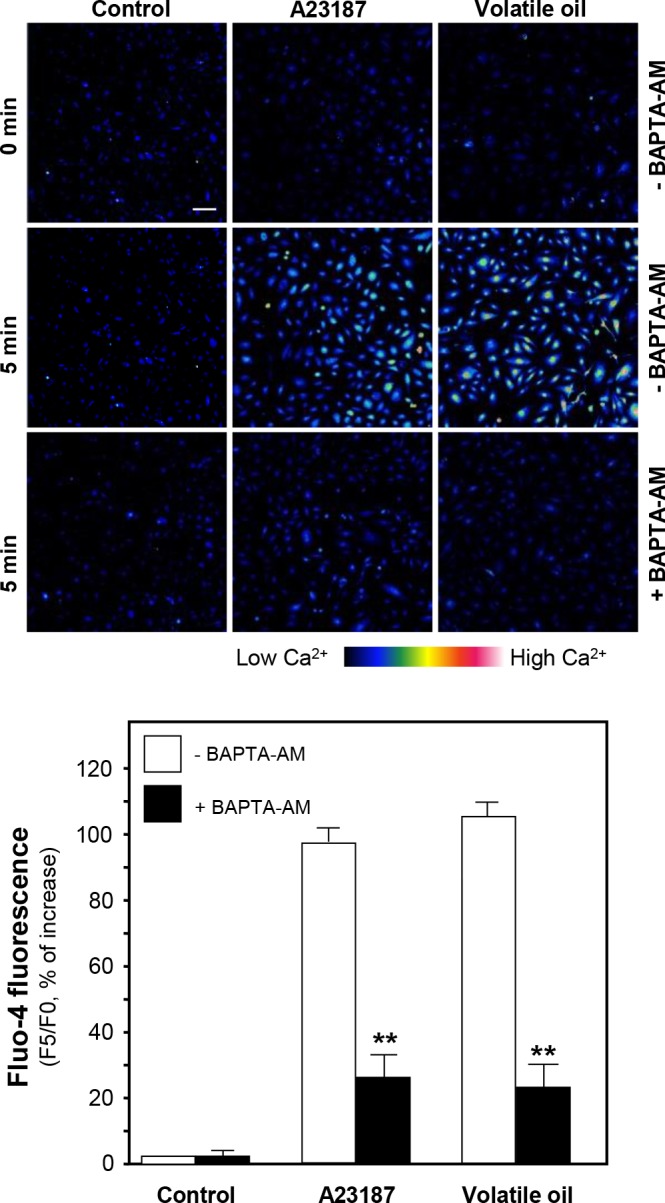
NRR volatile oil induces Ca^2+^ mobilization in HUVECs. Cultured HUVECs were labeled with fluorescent Ca^2+^ indicator Fluo-4 AM for 30 min. Fluorimetric measurement was performed after the treatment of NRR volatile oil (25 µg/mL), A23187 (1 µM, positive control) or control (untreated culture) (upper panel). Bar = 100 µm. Quantification of Ca^2+^ mobilization was displayed as a ratio of fluorescence intensity at 5 min (F5) to the control at time 0 (F0) in the cultures (lower panel). Mean ± SEM, *n* = 3, each with triplicate samples. **p<0.01.

**Figure 7:**
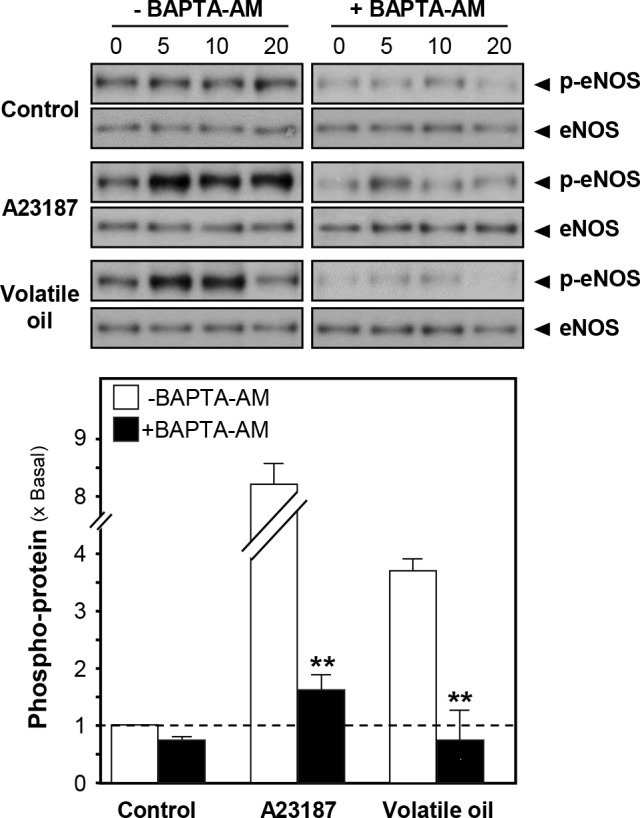
NRR volatile oil-induced eNOS phosphorylation is mediated by Ca^2+^/CaM pathway. Cultured HUVECs were pre-treated with serum free medium or BAPTA-AM (5 µM) for 3 hours, and treated with NRR volatile oil (25 µg/mL), A23187 (1 µM, positive control) or control (serum free medium) for different time points (0–20 min). Phospho-eNOS Ser^1177^ (~135 kDa) and total eNOS (~135 kDa) were revealed by using specific antibodies (upper panel). Quantification of phospho-eNOS protein (at 5 min) from the blot was calculated by a densitometer (lower panel). Data were expressed as x Basal where the control (untreated culture) was set as 1, Mean ± SEM, *n* = 4, each with triplicate samples. **p<0.01.

**Figure 8:**
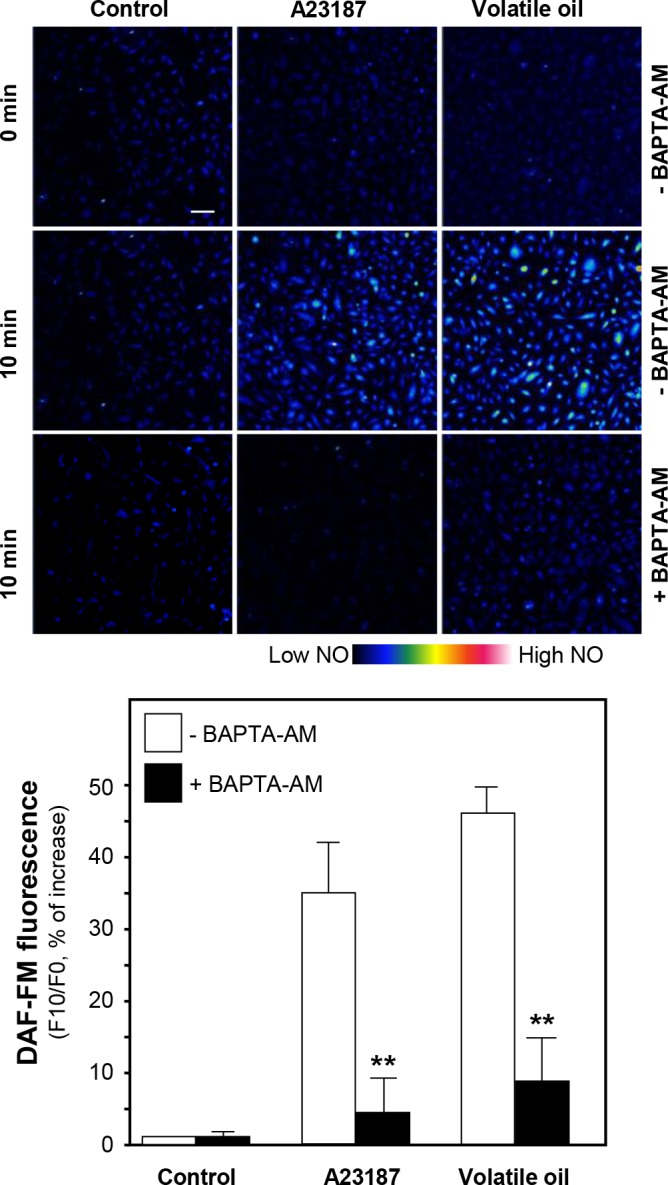
Volatile oil-induced NO production is blocked by BAPTA-AM. Cultured HUVECs were pre-treated with serum free medium or BAPTA-AM (5 µM) for 3 hours, and then labeled with fluorescent NO indicator DAF-FM DA for 30 min. Fluorimetric measurement was performed after the treatment of NRR volatile oil (25 µg/mL), A23187 (1 µM, positive control). The amounts of NO were evaluated by measuring the fluorescence intensity. Micrographs were taken by the confocal microscope. Bar = 100 µm (upper panel). Quantification of NO production was displayed as a ratio of fluorescence intensity at 10 min (F10) to the control at time 0 (F0) in the cultures (lower panel). Mean ± SEM, *n* = 3, each with triplicate samples. **p<0.01.

CaM kinase II is a serine/threonine-specific protein kinase that is regulated by the Ca^2+^/CaM complex. CaM kinase II could mediate the rapid activation of eNOS and vasodilation [16]. Thus, intracellular Ca^2+^ change and eNOS phosphorylation were investigated after treatment of NRR volatile oil in HUVECs. Fluo-4 AM, a Ca^2+^ indicator, was applied to monitor the change of Ca^2+^-induced fluorescence signal in HUVECs. The increase of Ca^2+^ level was found after treatment of NRR volatile oil (Fig. 6). Pre-treatment of Ca^2+^ chelator, BAPTA-AM, markedly suppressed Ca^2+^ increase, both in NRR volatile oil- and A23187-treated HUVECs (Fig. 6). Cultured HUVECs were pre-treated with BAPTA-AM for 3 hours, and then the amount of phosphorylated eNOS was determined after the treatment of NRR volatile oil. The pre-treatment of BAPTA-AM fully reduced the volatile oil-induced eNOS phosphorylation (Fig. 7). In addition, BAPTA-AM pre-treated HUVEC cultures were subjected to the measurement of NO production after treatment of NRR volatile oil. The volatile oil-mediated NO production was suppressed by pre-treatment of BAPTA-AM (Fig. 8). The blockage by BAPTA-AM was also applied to the case of A23187 in inducing eNOS phosphorylation and NO production (Figs. 7 and 8).

## Discussion

Cardiovascular disease accounts for the majority of morbidity and mortality in the world. Most forms of cardiovascular diseases involve atherosclerotic vascular change in the coronary, cerebral, renal and peripheral circulation leading to angina pectoris, myocardial infarction and stroke. Endothelial function is impaired in certain cardiovascular conditions including atherosclerosis [20]. Atherosclerosis impairs endothelium-dependent coronary vasodilation and thereby may predispose to vasoconstriction, a paradoxic vasoactive response that represents a fundamental defect in endothelial regulation of vascular tone [21]. Endothelial cells release vasoactive substances, e.g. NO, regulate vascular smooth muscles and trafficking blood cells [22]. Indeed, several cardiovascular drugs are targeting to NO-signaling. Here, we provided different lines of evidence to support the notion of NRR usage in traditional Uyghur medicine, which frequently was prescribed in herbal decoctions, e.g. Song Bu Li decoction [7, 9]. Song Bu Li decoction is very popular in Uyghur hospitals in Xinjiang of China for the treatment of cardiovascular diseases [9]. This decoction is composed of NRR water extract plus its volatile oil [6]. Having the isolated volatile oil from NRR, we demonstrated signaling triggered by NRR volatile oil in cultured human umbilical vein endothelial cells (HUVECs): (i) the activation of NO production and eNOS phosphorylation; (ii) the phosphorylation of Akt kinase; and (iii) the mobilization of intracellular Ca^2+^. These functions therefore fully supported the clinical usage of NRR and/or Song Bu Li decoction.

NO was synthesized in endothelium, and this resulted further relaxation of blood vessel [23]. NO inhibits the platelet activation, adhesion, secretion and aggregation. eNOS is responsible for NO synthesis. The activation of eNOS is triggered by phosphorylation, and Ser^1177^ appears to be the most important site for regulation [24]. Akt kinase is known to phosphorylate eNOS at Ser^1177^, resulting in activation of eNOS activity and regulation of vascular tone [25]. For example, the NO production in responding to shear stress was controlled by the Akt-dependent phosphorylation of eNOS in cultured HUVECs [15]. On the other hand, the phosphorylation and activation of eNOS at Ser^1177^ could be mediated by CaM kinase via the mobilization of Ca^2+^ [14, 26, 27]. By using various inhibitors here, the eNOS phosphorylation and NO production triggered by NRR volatile oil could be mediated by both Akt and CaM kinase activation. The complete blockage by a Ca^2+^ chelator, BAPTA-AM, in NRR-induced eNOS phosphorylation suggesting that Ca^2+^ mobilization could be the upstream of both signaling triggered by Akt and CaM kinases. Here, the NRR oil is believed to activate intracellular Ca^2+^ surge; however, whether the involvement of a cell surface receptor in this activation has not been revealed.

The inactivation of NO could be mediated by reacting with superoxide anion to form the potent oxidant peroxynitrite [28, 29]. Thus, reactive oxygen species (ROS) impairs endothelium-dependent relaxation via NO reduction [30]. Our recent report demonstrated that NRR volatile oil possesses strong protection effect against oxidative stress induced by ROS in cultured cardiomyocyte [10]. In this notion, the volatile oil might increase the bioavailability of NO by suppressing the intracellular ROS. It is believed that oxidative stress is important mechanism underlying various forms of cardiovascular disease, including atherosclerosis, myocardial ischemia-reperfusion injury [31].

Although NRR volatile oil can trigger beneficial pharmacological actions, the responsible active ingredients have never been identified. This is a problem not only for NRR but also for the majority of other herbal medicines. The major chemical components of NRR volatile oil are calarene, β-maaliene and aristolene [6, 10]; but their role in vascular functions has not been determined. Similar to NRR, many herbal extracts have been employed as a vasodilator. In Chinese herbal mixture, Fo Shou San is widely used in circulatory and cardiovascular disorders, and which triggers the vasodilation via eNOS [17]. Fo Shou San composed of Angelicae Sinensis Radix and Chuanxiong Rhizoma: both of these two herbs contained high amount of volatile oil. For example, ligustilide and butylidenephthalide were reported to have vasodilation effect on rat abdominal aorta [32]. Volatile oil is a common ingredient of many herbs, and the application of which is very restricted today, and therefore studies are needed to pave a direction in finding active ingredients from there.

## Conclusion

Our study focused on the vascular effect of volatile oil derived from NRR in HUVEC cells. The treatment of NRR volatile oil could increase the production of NO, an endothelium derived relaxation factor, in HUVECs. This induction could be mediated by phosphorylation of eNOS via Akt phosphorylation and intracellular Ca^2+^ elevation.

## Supporting Information

S1 Fig Effect of NRR volatile oil on the viability of HUVEC cells. Cultured HUVEC cells were treated with the dose of (3 to 100 µg/mL) of volatile oil for 24 hours. Cell viability and proliferation test (using the colorimetric MTT assay) was performed. Data were expressed as a percentage of increase as compared to the control (untreated culture), Mean ± SEM, *n* = 4, each with triplicate samples.
(TIF)
