# Supplementary figures and images for: The Volatile Oil of Nardostachyos Radix et Rhizoma Induces Endothelial Nitric Oxide Synthase Activity in HUVEC Cells
Source: PLoS One. 2015 Feb 2;10(2):e0116761. doi: 10.1371/journal.pone.0116761 (PMC4359165; doi:10.1371/journal.pone.0116761)

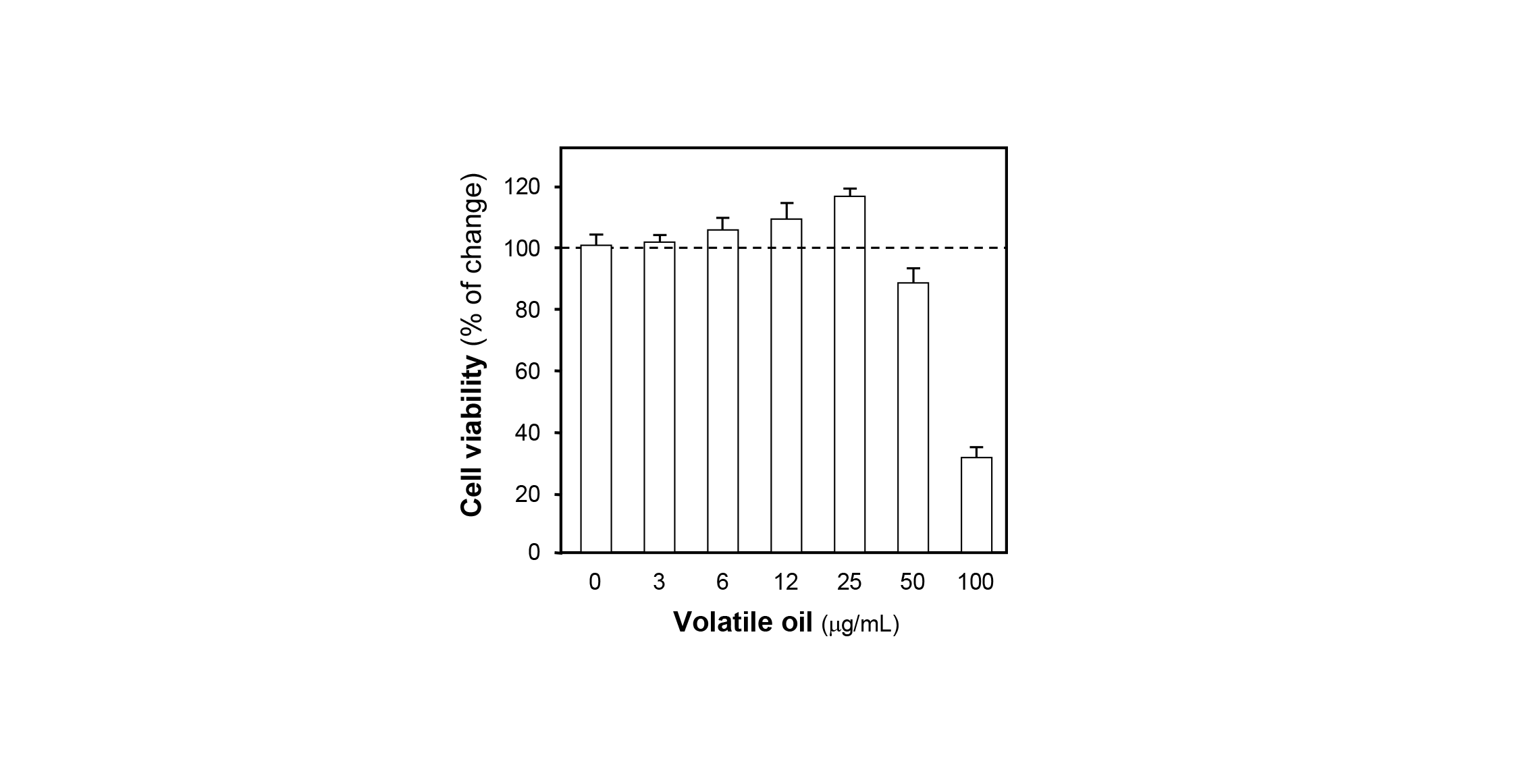

Supplement: S1 Fig — Cultured HUVEC cells were treated with the dose of (3 to 100 µg/mL) of volatile oil for 24 hours. Cell viability and proliferation test (using the colorimetric MTT assay) was performed. Data were expressed as a percentage of increase as compared to the control (untreated culture), Mean ± SEM, n = 4, each with triplicate samples. (TIF) [file pone.0116761.s001.tif]
